# Effects of Fermented Garlic Extract Containing Nitric Oxide Metabolites on Blood Flow in Healthy Participants: A Randomized Controlled Trial

**DOI:** 10.3390/nu14245238

**Published:** 2022-12-08

**Authors:** Ji Soo Baik, Ji Hong Min, Sung Min Ju, Jae Hyun Ahn, Sung Hwa Ko, Hyun Soo Chon, Min Sun Kim, Yong Il Shin

**Affiliations:** 1 Research Institute for Convergence of Biomedical Science and Technology, Pusan National University Yangsan Hospital, Yangsan 50612, Republic of Korea; zisoo@pusan.ac.kr; 2 Department of Rehabilitation Medicine, Pusan National University Yangsan Hospital, Yangsan 50612, Republic of Korea; papered@hanmail.net (J.H.M.); ijsh6679@gmail.com (S.H.K.); 3 Department of Pathology, College of Korean Medicine, Wonkwang University, Iksan 54538, Republic of Korea; biosci@naver.com; 4 Department of General Medicine, University of Medicine and Pharmacy Cluj-Napoca, 400347 Cluj-Napoca, Romania; ahn0217@naver.com; 5 Department of Rehabilitation Medicine, School of Medicine, Pusan National University, Yangsan 50612, Republic of Korea; 6 HumanEnos LLC., Wanju 55347, Republic of Korea; 119bio@naver.com; 7 Center for Nitric Oxide Metabolite, Wonkwang University, Iksan 54538, Republic of Korea

**Keywords:** fermented garlic extract, blood flow, blood pressure, nitric oxide

## Abstract

Aged or fermented garlic extract (FGE) is a natural remedy that improves vascular function through increasing vascular nitric oxide (NO) bioavailability. This is because nitrite (NO_2_^−^), a NO metabolite, can be produced through bioconversion with macrobacteria during the fermentation of foods like garlic. We aimed to evaluate the effects of NO_2_^−^ in FGE on blood flow (BF), blood pressure (BP), velocity of the common carotid artery (CCA) and internal carotid artery (ICA), regional cerebral BF (rCBF), and peripheral BF (PBF). The study was divided into two parts: (1) Thirty healthy adults were divided into FGE and placebo groups to compare BP and velocity of the CCA and ICA; and (2) Twenty-eight healthy adults were divided into FGE and placebo groups to compare rCBF and PBF and determine changes before/after ingestion. Significant changes were noted in BP and the velocity of both CCA 30–60 min after FGE ingestion. FGE ingestion resulted in significant increases in rCBF and increases in body surface temperature through alterations in PBF. No detectable clinical side effects were noted. Overall, oral administration of NO_2_^−^ containing FGE demonstrated acute positive effects in upregulating BF, including the CCA, BP, rCBF, and PBF. Follow-up studies with larger sample sizes and long-term ingestion may be needed.

## 1. Introduction

Cardiovascular disease (CVD) is the leading cause of mortality worldwide [1], and age is a major risk factor. CVD related mortality increases in older adults, due in large part to adverse changes occurring in arteries associated with vascular dysfunction. Age-related declines in cardiovascular functions may impair cerebral blood flow (BF) regulation, leading to the disruption of neuronal micro-environmental homeostasis [2]. As the brain requires a large amount of energy to sustain neuronal metabolism, cerebral BF is essential in maintaining normal brain functions. Moreover, the impairment of cerebrovascular or neurovascular functions can increase the incidence of neurological disorders, such as vascular cognitive impairment and Alzheimer’s disease [3].

Nitric oxide (NO) is a gaseous signal molecule that is generated from the endothelial cells and causes relaxation of vascular smooth muscle. In the aging process, progressive declines in NO production or NO bioavailability are associated with decreased antioxidants in the vessels [4]. Additionally, impaired NO bioavailability is characterized by disturbed vasodilator and anticoagulant function, increased inflammation, and a breakdown of barrier function, which leads to atherosclerosis formation [5,6]. This NO is produced through nitrate (NO_3_^−^)—nitrite (NO_2_^−^)—in the NO pathway [7,8,9]. Alternatively, when NO_3_^−^ is aged or fermented, it is converted to NO_2_^−^. NO_2_^−^ is converted to NO by combining with glutathione present in the human body, or combining with flavonoids derived from plants, or in response to intestinal microorganisms [7,8,9]. The NO_3_^−^ –NO_2_^−^– NO pathway is a major alternative source of NO and is essential for NO dependent physiological functions in the body [7,8,9]. In particular, NO_2_^−^ is emerging as an endogenous signaling molecule, with potential therapeutic implications for CVD [10,11,12].

Many plants contain NO_3_^−^ [13]. NO_3_^−^ can naturally change to NO_2_^−^ through digestion in the human body, but most of it is discharged without switching [14]. We hypothesized that nitric oxide-related effects would be greater in the body if plants were fermented and consumed in the NO_2_^−^ state. We previously developed a new bioconversion technique that allows for the production of high concentrations of NO_2_^−^ from various plant materials, including garlic, lettuce, and beans. This occurs during the fermentation process and requires the long-term stabilization of NO_2_^−^ in fermented fluid [15,16,17]. In earlier studies, fermented garlic extract (FGE) has shown to decrease BP in hypertensive rodent models through the activation of intracellular NO signaling in the artery [18]. Additionally, FGE attenuated monocrotaline-induced pulmonary hypertension by decreasing pulmonary endothelial injury via the NO-sGC-PKG pathway [19]. Direct application of FGE into the brain surface induced the upregulation of cerebral BF through activation of intracellular NO signaling [20].

In previous animal studies, we confirmed that FGE containing NO_2_^−^ may result in effective NO signaling in the vascular system. However, more research is needed to confirm the effectiveness and safety of FGE in humans. Therefore, in this study, we aimed to confirm whether FGE contributed to changes in BF in healthy adults, including regional cerebral blood flow (rCBF), carotid artery (CA) BF, peripheral blood flow (PBF), and BP and to confirm safety.

## 2. Materials and Methods

### 2.1. Preparation and Fermentation of Garlic Fermented Broth

The garlic fermented broth used in this study was supplied by HumanEnos (Wanju-gun, Jeonbuk, Republic of Korea) and was prepared similarly to previous studies [18,21]. The manufacturing process included first peeling the skin from raw garlic. The garlic was then washed, sterilized, and ground for 24 hours, after which, the mixture was diluted with water at a constant ratio (1:9 [*w/v*]). Activated *Bacillus subtilis* was then inoculated and aerobic fermentation was carried out at 37 °C for one month. Fermentation was stopped when NO_2_^−^ in the fermentation broth reached a concentration of 150 ppm or more. The supernatant was separated from the suspension by centrifuge, and the separated fermented garlic solution was concentrated using an evaporator to dry FGE up to at least 2,000 ppm of the concentration of NO_2_^−^ [18,19].

### 2.2. Determination of NO_2_^−^ Ions in the Garlic Fermentation Broth and NaNO_2_ Solution

NO_2_^−^ levels in the fermentation broth or dried FGE were quantified using the NO_3_^−^/NO_2_^−^ Colorimetric Assay Kit (Cayman Chemical Co., Ann Arbor, USA). The same amounts of reaction solution and Griess reagent (2.5% [*v/v*] phosphoric acid, 1% [*w/v*] sulfanilamide, and 0.1% [*w/v*] naphylethylenediamine) were mixed. After reacting at room temperature for ten min, the absorbance was measured at 540 nm using a UV spectrophotometer (Ultrospec 2100 Pro, Amersham Pharmacia Biotech, Cambridge, UK). In order to quantify the NO_2_^−^ amount in the garlic fermentation broth, NaNO_2_ (Sigma-Aldrich^®^ Co., St. Louis, MO, USA) as a standard material was diluted with distilled water. After reacting the diluted NaNO_2_ solution with Griess reagent, the standard curve was generated by using the UV spectrophotometer configured for UV absorbance measurements. NO_2_^−^ levels were then quantified by applying the absorbance value of garlic fermentation broth to the standard curve.

### 2.3. Determination of NO Release in Simulated Gastric Fluid

**Figure 1:**
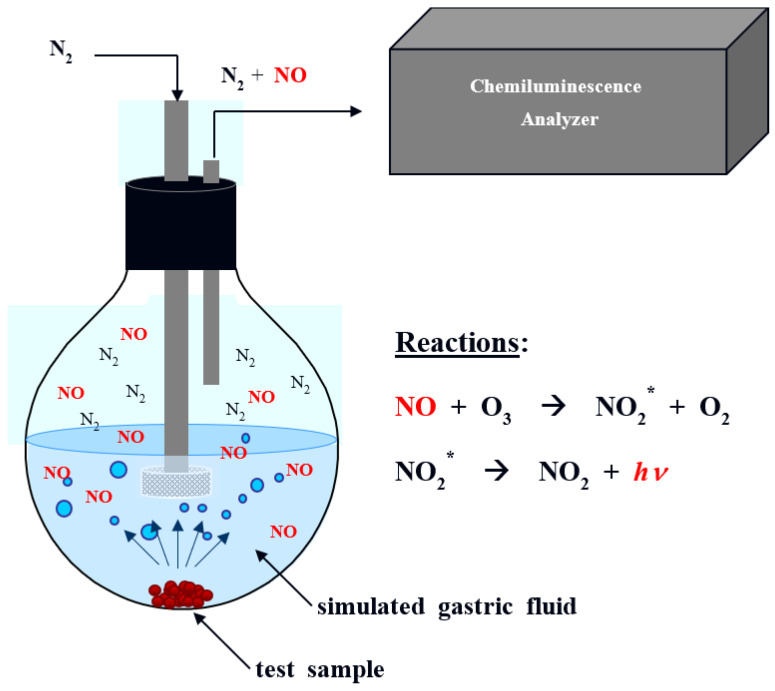
NO, nitric oxide; O_3_, ozone; NO_2_*, excited nitrogen dioxide; O_2_, oxygen; *hv*, photon; Schematic illustration of the chemiluminescence technique for measurement of NO gas generated from the test sample in simulated gastric fluid (pH 2). NO reacts with O_3_ to create NO_2_*, and NO is quantified by measuring the *hv* emitted when NO_2_* converted to stable NO_2_.

NO generation profiles from FGE in simulated gastric fluid (SGF) were collected using a Sievers 280 chemiluminescent NO analyzer (Boulder, CO, USA). SGF is composed of 50 mM HCl, 75 mM NaCl, and 13 mM KCl (pH 1.3). The instrument was calibrated with an atmospheric sample that had been passed through an NO zero filter and a 24.1 ppm NO gas standard (balance N_2_). One FGE tablet was finely ground, dissolved in phosphate buffered saline (1 mL), and then immersed in deoxygenated SGF at 37 °C. Liberated NO was provided from the SGF to the analyzer with a stream of N_2_ bubbled into the solution at a flow rate of 80 mL/min. NO was detected via a chemiluminescent reaction with ozone (Figure 1). As control, one tablet of Neo40^®^ (Human^n^, Austin, TX, USA), which is a representative NO-increasing supplement, was finely ground, dissolved in the buffer (1 mL), and put in the SGF to quantify the amount of NO release.

### 2.4. Formulation of FGE Tablets

One tablet of encapsulated FGE consisted of 180 mg of dried FGE containing 7 mg of NO_2_^−^ and 270 mg of other food grade ingredients. The dose of NO_2_^−^ ions in one FGE tablet was determined based on our previous animal study where the oral, single administration of FGE solution (10 mg of NO_2_^−^/mL) showed dose-dependent decrease in systolic BP in hypertensive rats [18]. The dose was also determined by other clinical studies that demonstrated decreased BP due to inorganic NO_2_^−^ supplements without clinical side effects [22,23]. FGE and placebo groups were administered single-dose 450 mg encapsulated FGE and 450 mg placebo tablets, respectively. The placebo tablet was prepared with a food ingredient that was harmless to the human body that did not contain garlic, and its weight and shape were almost the same compared with the FGE tablet.

### 2.5. Clinical Study

#### 2.5.1. Experimental Design and Participants

This study was conducted from October 2020 to March 2021. This study was approved by the Ethics Committee of Pusan National University Yangsan Hospital (approval no. 04-2020-038, 04-2020-036), and registered on ClinicalTrials.gov (accessed on 1 November 2022) (NCT05349604, NCT05349253).

**Figure 2:**
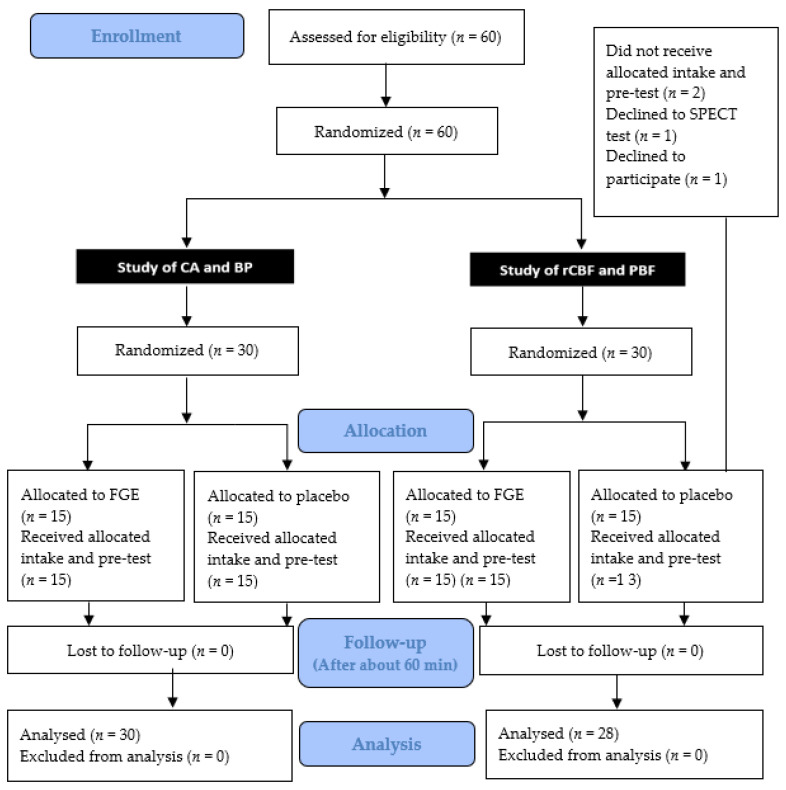
Flowchart of the study progress according to the CONSORT guidelines.

Informed consent was obtained from all interested and eligible participants. A total 60 participants were initially included in the experiment and were divided into each study: (1) a study of CA and BP (04-2020-038, NCT05349604) and (2) a study of rCBF and PBF (04-2020-036, NCT05349253). Two participants were ultimately excluded, as one declined to participate and one canceled the evaluations. Therefore, 58 participants were enrolled in this experiment. The characteristics of the participants (*n* = 58) in each experimental group are summarized in Table 1. Figure 2 is an overview of the study progress according to the CONSORT guidelines.

This experiment was an investigator-led, randomized, double-blind, placebo-controlled study. We determined the experiment group by: (1) screening for BP; (2) surveying for demographic data, including sex, age, height, and weight; (3) determining the past medical history and concomitant diseases present; (4) smoking and drinking history intake; (5) drug administration history intake; and (6) fertility, pregnancy, and lactation history.

We recruited adults aged ≥19 years who had not participated in any other clinical trials in the past 3 months. Pregnant women, individuals taking medications or food that can affect BP, and individuals with severe comorbidities, such as hypertension, cardiovascular disease, autoimmune diseases, and liver or kidney disease were excluded from the study.

#### 2.5.2. Doppler Ultrasonography (CA and BP)

Thirty healthy individuals were recruited and equally and randomly divided into FGE and placebo groups. On the day of the test, participants visited the Department of Neurology, Pusan National University Yangsan Hospital and had carotid Doppler ultrasounds performed to measure the changes in the velocity of the CA. The CA velocity was measured by subdividing the artery into the common carotid artery (CCA) and internal carotid artery (ICA). The BP was measured using a BP measurement device (BPBIO320, Inbody, Seoul, Republic of Korea).

**Figure 3:**
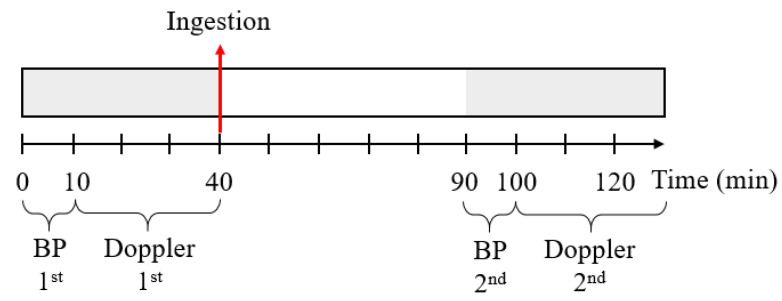
Timeline of the first part of the clinical study to measure carotid artery velocity and blood pressure (BP).

Before ingesting FGE or placebo tablets, the Doppler ultrasounds of the bilateral CA and BP measurements were performed to get baseline values. After, tablets were administered to the participants, and CA velocity and BP measurements were performed again approximately 30–60 min after ingestion [24] (Figure 3).

#### 2.5.3. Measurement of rCBF and PBF

**Figure 4:**
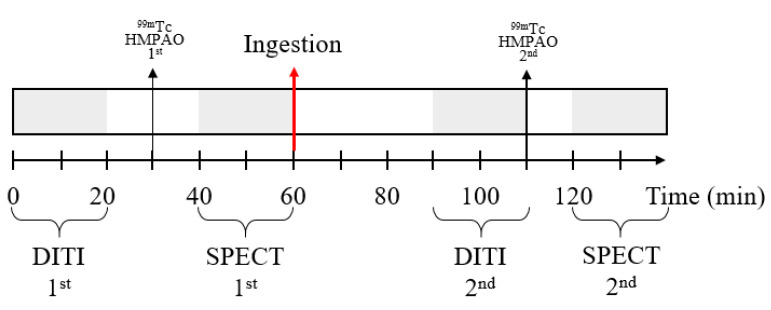
Timeline of the second part of the clinical study to measure rCBF and PBF. SPECT, single photon emission computed tomography; DITI, digital infrared thermographic imaging; rCBF, regional cerebral blood flow; PBF, peripheral blood flow.

Twenty-eight healthy individuals were recruited and were randomly divided into groups of 15 and 13 participants for the FGE and placebo groups, respectively. Before ingesting the FGE or placebo tablet, 28 participants were examined using single photon emission computed tomography (SPECT, General Electric NM 830 SPECT, USA) and digital infrared thermographic imaging (DITI, Iris-XP, Medi-core, Seoul, Republic of Korea) scanning. Then, the test product was orally administered to the participants. Thereafter, they underwent SPECT and DITI measurements again around 30–60 min after ingestion (Figure 4). The primary outcome assessment was made by rCBF using SPECT [25,26]. Changes in rCBF were measured through Tc-99 m HMPAO SPECT imaging, which was then converted into a Z-score using Tc-99 m HMPAO SPECT [27]. The standard set of the volume that defines each cortex area was analyzed by a *p* value < 0.0001 (cluster size 50 voxel), based on the Automated Anatomical Labeling Atlas [28]. In this study, the body surface temperature (BST) test was performed to confirm the PBF. The BST was measured using DITI (Iris-XP, Medi-core, Seoul, Republic of Korea).

### 2.6. Safety Variable Analysis

Safety evaluations were conducted on all participants who participated in the study and participants who ingested the product at least once. The NCI Common Terminology Criteria for Adverse Events (CTCAE, ver4.0) was utilized, with responses of “none (0)”, “mild (1)”, “moderate (2)”, and “severe (3)”.

### 2.7. Statistical Analysis

The data were analyzed within each study, and no statistical analyses were performed between the two studies. The Wilcoxon signed rank test is a nonparametric statistical hypothesis test used to evaluate whether population means differ in rank by comparing repeated measures of two related, matched, or single samples. Efficacy was evaluated using Wilcoxon signed rank tests and before-after differences were evaluated using Mann–Whitney U tests. Ranked analysis of covariance (Quade’s test) was also used to control for baseline differences in age and covariates as well as to compare the outcome before and after ingestion. A *p*-value of <0.05 was considered to be statistically significant, and statistical analyses were performed using R statistical software (version 4.0.3; The R Foundation). The SPECT image data were analyzed using a 3D voxel-based statistical analysis (SPM) procedure and an ROI-based method (*p _FWE-corr_* < 0.001, uncorrected for multiple comparison with cluster extent threshold Ke = 50 voxels).

## 3. Results

### 3.1. Induction of NO from FGE under Artificial Gastric Juice

**Figure 5:**
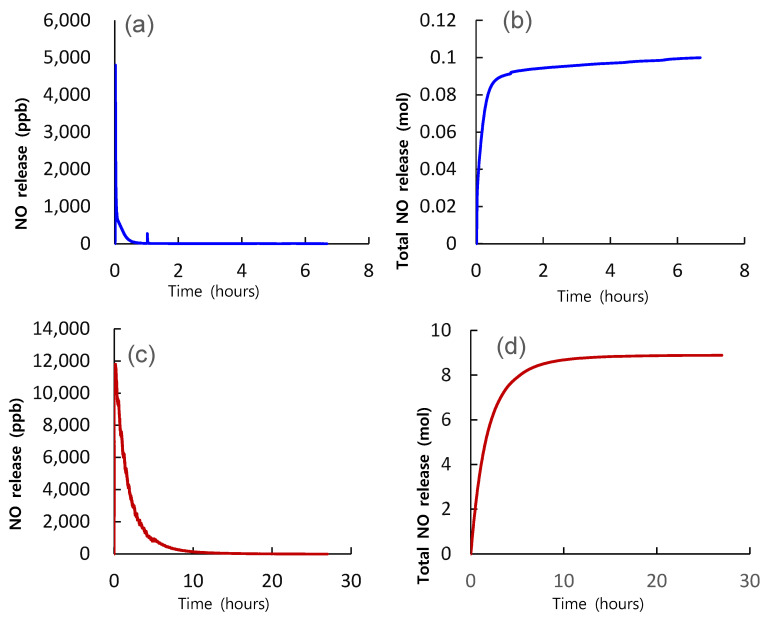
Representative line histograms showing time-dependent changes of NO release of the single FGE tablet and Neo40^®^ tablet on the artificial gastric juice (pH 1.5), respectively. Panels (**a**,**b**) present the data of Neo40^®^ tablets, while panels (**c**,**d**) present the data of FGE tablets. Panels (**a**,**c**) show the concentration (ppb) of NO over time, while panels (**b**,**d**) present the mass (mol) of NO over time.

Compared to the Neo40^®^ tablet, the FGE tablet in this study had a higher amount of NO release and a longer duration of NO release time in the artificial gastric juice (pH 1.5). Based on the serving size, the total mass of NO produced by one tablet of Neo40^®^ was approximately 0.1 mol, and the total mass of NO produced by one tablet of FGE was approximately 8.9 mol. Moreover, the initial flux of NO was approximately 4800 and 11,800 ppb in the Neo40^®^ and FGE tablets, respectively. In addition, the durations of NO until the time when NO release was finished were < 6 h and 24 h for the Neo40^®^ and FGE (Figure 5) tablets, respectively.

### 3.2. Changes in BP and CA Velocity

Thirty participants completed the study and did not drop out. The results of the test of equal variances for the two sexes were F = 0.083, *p* = 0.934. The difference between the FGE and placebo groups had a *p*-value > 0.05, indicating that there was no statistically significant difference. Therefore, no significant differences existed in the sex distribution between the two groups, indicating that homogeneity was secured. Additionally, the FGE and placebo groups had a mean (±standard deviation) age of 61.33 (±4.85) and 61.20 (±12.91) years, respectively. The results of the test of equal variances for the two groups was F = 1.466, *p* = 0.217. The difference between the FGE and placebo groups had a *p*-value > 0.05, indicating that there was no statistically significant difference in the age distribution between the two groups and that homogeneity was secured.

Table 2 shows the result of the changes in BP over time in each group. In the placebo group (*n* = 15), baseline systolic and diastolic BPs were 133.4 ± 23.83 mm Hg and 84.20 ± 17.68 mm Hg, respectively, and the baseline heart rate was 80 ± 10 bpm. Slight reductions in systolic (9.20 mm Hg) and diastolic (9.07 mm Hg) pressures were noted after ingestion of the placebo tablet; however, the changes were not statistically significant. In contrast, in the FGE group (*n* = 15), the average baseline systolic and diastolic BPs were 124.0 ± 13.7 mm Hg and 79.4 ± 12.3 mm Hg, respectively. A marked reduction of systolic (16.93 mm Hg) and diastolic (12.34 mm Hg) BPs 30 min after the ingestion of the FGE tablet was observed, which was significantly statistically different than the before values (*p* = 0.001).

**Figure 6:**
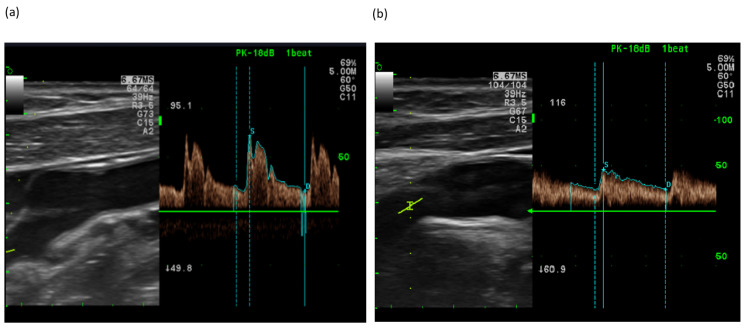
Representative Doppler ultrasound images illustrating changes in blood flow in the common carotid artery before (**a**) and after (**b**) the ingestion of a fermented garlic extract tablet.

The CA velocity of the bilateral CCA and ICA was measured by Doppler ultrasound before and after ingestion of the test products. In the placebo group, the peak systolic flow velocity (Psv) of the right CCA was 22.2 ± 2.2 cm/sec before and 22.4 ± 4.4 cm/sec 30 min after taking the placebo tablet. In the FGE group the Psv of the right CCA was 24.4 ± 2.2 cm/sec before and 19.9 ± 4.4 cm/sec 30 min after taking the FGE tablet (Figure 6).

According to the statistical analysis, the treatment with the FGE tablet in the FGE group caused a marked reductions in the Psv and peak diastolic flow velocity (Edv) in right the CCA (Z = −2.413, *p* = 0.016, Z = −3.114, *p* = 0.002), right ICA (Z = −2.480, *p* = 0.013), and left CCA (Z = −2.204, *p* = 0.028, Z = −2.240, *p* = 0.025). In the placebo group, the placebo treatment did not induce a significant change in either the Psv or Edv of the CCA and ICA (Table 3).

Significant differences between the FGE group and the placebo group regarding the amount of change before and after ingesting FGE were found in systolic BP (Z = −2.263, *p* = 0.024) and diastolic BP (Z = −2.637, *p* = 0.008). And Significant differences between the FGE and placebo groups regarding the changes in Psv or Edv before and after ingesting FGE were found in the right CCA Edv (Z = −2.167, *p* = 0.030) and left CCA Edv (Z = −2.065, *p* = 0.039) (Table 4).

### 3.3. Changes in rCBF and PBF

A total of 30 participants were initially included; however, one declined to be evaluated, and the other cancelled the scheduled participation. Therefore, the results were analyzed for a total of 28 participants. The 15 FGE group participants were composed of eight males and seven females, while the 13 placebo group participants were composed of three males and ten females. The average age of the FGE and placebo groups was 54.3 ± 10.9 years and 55.9 ± 13.9 years, respectively.

**Figure 7:**
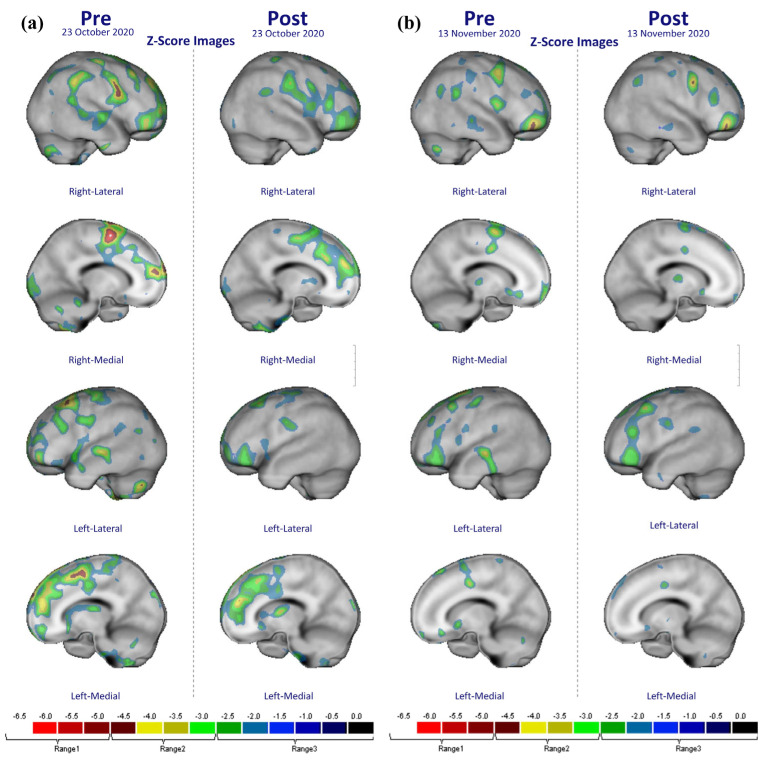
Diagrams showing regional cerebral blood flow in the brain of the fermented garlic extract (**a**) and placebo tablet ingestion groups (**b**). The closer the z-score is to 0 (black color), the closer it is to the normal value.

Changes in rCBF in the brain were measured using the Tc-99m HMPAO SPECT imaging technique before and after taking placebo or FGE tablets. A *p _FWE-corr_* of <0.001 was considered to be statistically significant based on SPM analysis (50 voxel). The post-test results, excluding the pre-effects of the FGE and placebo groups, were compared. Regarding rCBF, statistically significant trends were found in the right and left frontal cortex (Brodmann area 6, Brodmann area 9, and 10) and the right parietal cortex (Brodmann area 2) (Table 5). Figure 7 shows a statistically significant increase in the rCBF in the brains of the FGE group compared with the placebo group. However, no statistically significant changes in rCBF were observed in the placebo group.

**Figure 8:**
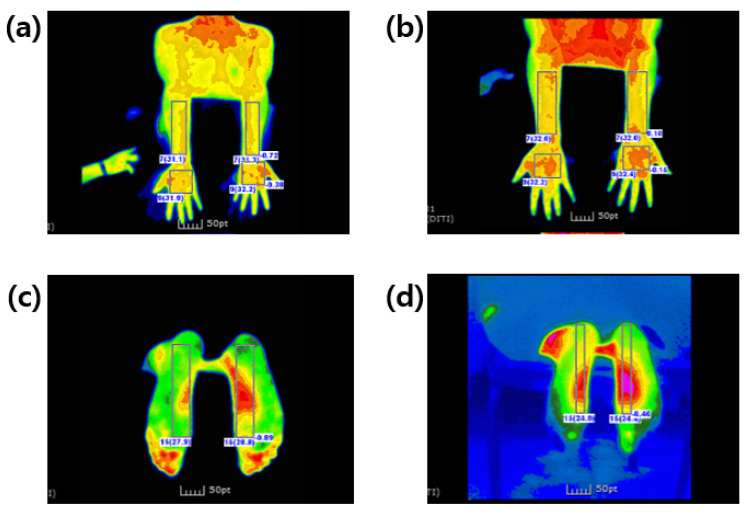
Representative photographs showing an increase in body surface temperature before (**a**,**c**) and after (**b**,**d**) FGE tablet ingestion. Images (**a**,**b**) show the posterior forearm, and images (**c**,**d**) show the plantar area.

Statistically significant differences in BST between baseline and 30 min after ingestion of FGE tablets were noted in the following extremities: posterior forearm (F = 5.706, *p* = 0.025), palm (F = 4.864, *p* = 0.037), and plantar (F = 6.534, *p* = 0.017) areas. However, significant changes were not observed in these areas in the placebo group (Figure 8).

### 3.4. Evaluation of Adverse Side Effects

Safety evaluations were conducted on all participants who ingested FGE or placebo tablets at least once. The NCI Common Terminology Criteria for Adverse Events (CTCAE, version 4.0) was utilized, with responses of “none (0)”, “mild (1)”, “moderate (2)”, and “severe (3)”. No adverse events, such as pain, low-temperature burns, skin redness, itching, photosensitivity, anxiety, fever, and headache, were observed.

## 4. Discussion

In the present blinded, placebo-controlled study, we evaluated the acute effects of FGE tablets containing 7 mg of inorganic NO_2_^−^ ions on BP, velocity of the CA, PBF, and rCBF in 58 adults. A major finding of this study included decreases in both systolic and diastolic BP 30 min after ingestion of the FGE tablet. Furthermore, systolic and diastolic blood velocities in the CCA were significantly reduced following ingestion of the FGE tablet. Additionally, FGE intake resulted in statistically significant increases in the BST in extremities, suggesting that vasodilatation of the peripheral vasculature on the skin may be caused by FGE intake. Similar to our results, a single dose of NO supplementation made of sodium NO_2_^−^ (20 mg) and phytochemicals resulted in a significant decrease of about 6 mm Hg in the resting systolic and diastolic BP, and also improved vascular stiffness and endothelial function in patients with hypertension [29]. The single oral dose of 80 mg sodium NO_2_^−^ led to a significant, asymptomatic systolic BP drop of 10/6 mm Hg, with no effect on diastolic pressure. Plasma NO_2_^−^ levels increased to 3–4 μM, or were approximately ten times higher than the normal steady state within one hour after dosing [30]. Recently, four meta-analyses and two original studies have demonstrated direct clinical evidence for the therapeutic effects of garlic extract in hypertension and in endothelial dysfunction in patients with diabetes [31,32,33]. Aged garlic supplementation can cause an approximately 4 mm Hg and 3 mm Hg decrease in systolic and diastolic BP, respectively, compared with controls [33].

Furthermore, chronic oral sodium NO_2_^−^ therapy (40 mg/three times daily) has been shown to significantly lower systolic, diastolic, and mean arterial pressures, but tolerance has been observed after 10–12 weeks of therapy in adults with hypertension and metabolic syndrome. Significant improvements in the intima-media thickness of the CA and trends toward improvements in flow-mediated vasodilation of the brachial artery and insulin sensitivity have been reported [23]. In a clinical trial, sodium NO_2_^−^ increased plasma NO_2_^−^ and was well tolerated. Additionally, brachial artery flow-mediated dilation (endothelial function) was increased 28% versus baseline after NO_2_^−^ supplementation (*p* < 0.05) [11]. A recent meta-analysis revealed that inorganic NO_3_^−^ intake was found to significantly reduce resting BP (systolic BP: −4.80 mmHg, diastolic BP: −1.74 mmHg), improve endothelial function (flow-mediated dilatation: 0.59%, *p* < 0.0001), reduce arterial stiffness (pulse wave velocity: −0.23 m/s; augmentation index: −2.1%,), and reduce platelet aggregation by 18.9% [34].

However, there are also differences between previous studies and this study. The first difference is that previous studies used much higher content of NO_2_^−^ capsules. The reason why a similar effect could be obtained despite the use of a tablet with a much lower NO_2_^−^ content than in previous studies is considered to be related to the results presented in Section 3.1. In the result, compared to the Neo40^®^ tablet, the FGE tablet in this study showed a longer amount of production and a longer stabilization period on the base of one serving size. A correct reason for a significant difference of NO production between the FGE and the Neo40^®^ under simulated gastric acid is not clear at this point Under acid juice, nitrite is largely reduced to NO in the presence of endogenous reductants, such as thiocyanate and ascorbic acid, whereas in the absence of these species, just 1% is converted to NO [35]. Hirota and Takahama (2014) [36] have reported that polyphenols in apple juice increase NO production from nitrite in acidic buffer solution (pH 2.0). Rocha and colleagues (2009) [37] have demonstrated that the degree of protonation to NO of nitrite from polyphenol/nitrite mixture under acidic juice (pH 2.0) is differs largely according to the type of polyphenol or concentration of polyphenol. For example, Epicatechin-3-O-gallate produces the highest NO production among the polyphenols tested in the study. Furthermore, in vivo NO production induced by the consumption of lettuce in the stomach of healthy volunteers is also different according to ingestion of different polyphenol-containing dietary foods such as apple, berries, cherries, black tea, and red wine 15 min after ingestion of lettuce. Collectively, considering these previous results showing the contribution of polyphenol or reductants to the reduction of nitrite to NO in an acidic medium, it can be postulated that different compositions of polyphenol or reductants are the reason for a significant difference of NO production between the FGE and the Neo40^®^ under the simulated gastric acid. Further research demonstrating the possible mechanisms of NO production from FEG tablets in the human body is warranted. In addition, another difference is that previous studies were focused on patients. We also plan to conduct follow-up studies using the FGE tablets in patients.

Our previous animal study [18] demonstrated that a single oral administration of FGE (10 mg of NO_2_^−^/mL) causes a decrease in BP in hypertensive rats, with a short half-life characterized by the peak effect at 30 min and recovery within 2–3 hours after feeding. Moreover, acetylcholine or sodium nitroprusside-induced vasodilatation was more augmented in the thoracic aortic strip of hypertensive rats who were given FGE for 12 days. The chronic feeding of FGE solution resulted in the increased expression of PKG and eNOS proteins in aortic tissue, which was prevented by pretreatment with soluble guanylate cyclase inhibitor (ODQ). In our recent ex vivo study, direct application of FGE with 20 ppm of NO_2_^−^ into the aortic ring of normotensive rats caused dose-dependent vasodilation. There was a partial inhibition of FGE-induced aortic vasodilatation caused by HXC, a NO scavenger and sodium pyruvate, an H_2_O_2_ scavenger, and total suppression of the vasodilation caused by ODQ, an sGC (unpublished data). The chemiluminescent detection method revealed that a marked increase in NO gas was detected when FGE was added into artificial gastric acid, indicating that NO_2_^−^ in FGE was protonated to NO. Collectively, present and previous results strongly suggest that the possible underlying mechanism of FGE with NO_2_^−^ is the activation of intracellular molecular events for eNOS and the sGC-cGMP-PKG pathway in the vascular smooth muscle. Additional modulations of intracellular molecular events associated with oxidative stress and inflammation by NO_2_^−^ ions also can restore vascular functions in aging vessels. For example, inorganic NO_2_^−^ can inhibit mitochondrial reactive oxygen species or oxidative stress that decreases endothelial function with aging. Sodium NO_2_^−^ supplementation can reduce blood concentrations of dehydroascorbate, the oxidized form of the superoxide-scavenging metabolite ascorbate, and inflammatory markers [11,38,39].

Our secondary major outcome was changes in rCBF, which showed statistically significant increases in the right and left frontal lobe and right parietal lobe following the ingestion of FGE. To the best of our knowledge, this is the first study to show increases in rCBF in local cortical areas following acute ingestion of NO_2_^−^ supplementation. A previous study observed no changes in the velocities in the large diameter of the middle cerebral artery measured by transcranial doppler ultrasound during continuous infusion of sodium NO_2_^−^ (0.6 mg/kg/h) [40]. In rats, intravenous infusion of NO_2_^−^ caused rapid increases in both NO production, by reduction of NO_2_^−^ in the red blood cells, and cerebral BF measured by laser Doppler flowmetry, which was suppressed by the inhibition of NO synthesis by L-NAME [41]

Supplementation of beet juice containing high NO_3_^−^ levels (12.4 mmol) in older individuals (aged > 70 years) for four days did not alter global cerebral perfusion, but did lead to increased resting regional cerebral perfusion within the bilateral white matter of the frontal lobes. However, no significant changes were noted in CBF in the cerebral cortex proper [42] using perfusion MRI. According to Wightman and colleague [43], near-infrared spectroscopy (NIRS) showed no changes in total hemoglobin during the abortion period. However, a significant transient increase of total hemoglobin, an index of cerebral BF, was noted during the initial cognitive task following ingestion of beetroot juice (~5.5 mmol of NO_3_^−^). The fMRI study demonstrated no basal changes in BF, but increased BOLD responses were observed in the visual cortex during visual stimuli following oral sodium NO_3_^−^ (0.1 mmol/kg/day) administration for three consecutive days [44]. Considering previous clinical results and our results, inconsistent effects of NO_2_^−^ or NO_3_^−^ supplementation on cerebral BF under resting conditions have been observed. These may primarily be due to the strong autoregulation mechanisms of cerebral BF, in place to minimize any changes in the cerebral circulation CBF under various physiological conditions. Various measurement or analysis methods for changes in CFG may also contribute to the inconsistencies.

In this study, the FGE group showed a significant improvement in the right frontal cortex (Brodmann area 6, Brodmann area 9, and 10) and right parietal cortex (Brodmann area 2) compared to the placebo group. Brodmann area 6 is mainly a role in planning a complicated and coordinated movement [45], and Brodmann areas 9 and 10 are mainly involved in functions such as short-term memory, space memory, calculations, language fluency, and problem solving [46,47,48]. Brodmann area 2 belongs to corresponds to the primary body sensory cortex, which is a major sensory receiving area for tactile sense [49]. As there was no study of cortex areas, in which the blood flow was changed after ingestion of FGE or garlic, it is difficult to compare our study results. However, the previous NO study reported that NO significantly increased the blood flow of the frontal cortex through vascular extensions [50]. In addition, previous studies that investigated the change in rCBF using low-level laser therapy (LLLT) reported that LLLT increased the cerebral blood flow of the frontal cortex and prefrontal cortex through increasing the NO level [51,52,53]. Based on these previous studies, we speculate that the reason for the increased rCBF in Brodmann areas 6, 9, and 10 included in the prefrontal cortex in this study may be the effect of NO. However, it is difficult to be certain about the effect on the right parietal cortex.

Garlic (*Allium sativum* L.) has been widely utilized as an important natural health remedy in improving various diseases in many ancient civilizations [54]. Functional sulfur-containing components presented in garlic include alliin, allicin, sulfides, diallyl trisulfide, and S-allyl-cysteine (SAC) [55]. Raw garlic extract (RGE) basically contains alliin, and when garlic is cut and the parenchyma is broken down, it is converted into allicin by the allinase enzyme. Several other organosulfur compounds, such as N-acetylcysteine, SAC, and S-ally-mercapto cysteine, are derived from alliin through aging or fermentation. In summary, it is known that allicin is a representative component in RGE, and SAC is a representative component in aged garlic extract (AGE) or FGE. Allicin helps with protein interaction and antioxidant activity, and SAC is known to improve antioxidant, anti-inflammatory, regulated redox, pro energetic, anti-apoptotic, and anticancer activities as well as signaling capacities [54,55]. Each company uses different garlic conditions, aging, fermentation, and processing methods. Thus, the ratio of compounds may vary. Previous studies have observed the highest allicin content (0.27%) in RGE, recommending the use of RGE rather than AGE [56]. However, many studies have demonstrated the effectiveness of FGE or AGE. The reason for this is considered to be that allicin, the main component found in fresh raw garlic and garlic powder, is highly volatile and unstable, and SAC, the main active compound, is highly stable [57].

However, in the FGE used in this study, alliin was detected at approximately 0.008 mg, and allicin and SAC were not detected [18]. Therefore, the intrinsic function of garlic compounds and their synergy with NO are considered important. For example, single dose of AGE temporarily increased production of NO metabolites in the plasma by increasing cNOS activity within 1 h of administration to mice [58]. Garlic extract (3–500 microg/mL) or allicin, major metabolites, produced dose- and NO-dependent relaxation under intact endothelium in rat pulmonary arteries [59,60]. Therefore, the action of vascular dilatation through the endothelial NO signaling of major garlic phytoconstituents could be due to the synergism of NO_2_^−^ induced changes in peripheral and central vascular function in this study. Therefore, it is difficult to ignore the synergistic effect with NO in supplements using garlic.

NO_2_^−^ ions can be produced as an intermediate through either nitrogen fixation or denitrification [61,62]. Denitrifying bacteria convert NO_3_^−^ into NO_2_^−^ using NO_3_^−^ reductase in the soil or fluid to make atmospheric nitrogen. Many vegetables, such as spinach, lettuce, celery, and red beets, are good sources of NO_2_^−^ or NO_3_^−^ for humans. During the fermentation process of these vegetables through denitrifying bacteria, preconverted, or natural NO_2_^−^, is made and then rapidly converted into NO_3_^−^ ions [63]. Vegetables are chewed in the oral cavity, and some are fermented by oral microorganisms. Through such a process, some NO_3_^−^ ions may be converted to NO_2_^−^, some go through digestive organs, and change into NO_2_^−^ or NO via fermentation by intestinal microorganisms in the stomach [64]. This natural NO_2_^−^ has been used as a natural curing agent for meat. The production of high concentrations of NO_2_^−^ in the FGE used in these studies may have been due to the aerobic denitrification or nitrification processed of bacillus strains [61]. However, compared with those of conventional fermentation procedures, the fermentation technique used in this study provided long-term stability of NO_2_^−^ ions in aqueous fermented solution.

The present study has several limitations. First, even though the data were obtained in a double-blind, randomized controlled study, this was a pilot study with a limited number of participants. Therefore, the findings may not easily be extrapolated to larger populations. Second, this study only looked at the short-term effects of an FGE tablet without a systematic strategy. Third, quality-of-life and hematological parameters in participants were not fully evaluated, and these parameters could have affected the clinical results. Fourth, serum measurements of NO or NO metabolites were not performed. Therefore, whether the FGE supplementation increased concentrations of NO in participants in unknown. However, based on previously published data that described sodium NO_2_^−^ supplement use and our clinical findings, FGE may increase NO production and availability. Further studies on the temporal profiles of NO metabolites in the blood following the ingestion of an FGE tablet may be needed.

## Figures and Tables

**Table 1:** Characteristics of participants.

Experiment Set	Groups	Age	Sex		Total Number
		M ± SD	Male	Female	
Study of CA and BP (04-2020-038, NCT05349604)	FGE	61.33 ± 4.85	8	7	15
	Placebo	61.20 ± 12.91	4	11	15
Study of rCBF and PBF (04-2020-036, NCT05349253)	FGE	54.27 ± 10.91	8	7	15
	Placebo	55.92 ± 13.91	3	10	13

CA, carotid artery; BP, blood pressure, FGE, fermented garlic extract; rCBF, regional cerebral blood flow; PBF, peripheral blood flow; M, mean; SD, standard deviation.

**Table 2:** Comparison of changes in arterial BP before and after FGE intake.

	Systolic BP				Diastolic BP			
	before	after	Z	*p*	before	after	Z	*p*
FGE	124 ± 13.76	107.07 ± 15.29	−3.325	* 0.001	79.47 ± 12.30	67.13 ± 12.47	−3.355	* 0.001
Placebo	133.40 ± 23.83	124.20 ± 26.19	−1.665	0.096	84.20 ± 17.68	75.13 ± 11.77	−1.877	0.061

* *p* < 0.05, Data are expressed as mean ± standard deviation. BP, blood pressure; FGE, fermented garlic extract.

**Table 3:** Comparison of CABF velocity before and after tablet ingestion.

		Right				Left			
		CCA		ICA		CCA		ICA	
		Psv	Edv	Psv	Edv	Psv	Edv	Psv	Edv
FGE	Z	−2.413	−3.114	−2.480	−1.819	−2.204	−2.240	−1.822	−0.377
	*p*	* 0.016	* 0.002	* 0.013	0.069	* 0.028	* 0.025	0.068	0.706
Placebo	Z	−1.540	−1.334	−0.385	−0.350	−0.974	−0.286	−0.472	−1.159
	*p*	0.124	0.182	0.701	0.726	0.330	0.775	0.637	0.246

* p < 0.05. CCA, common carotid arteries; ICA, internal carotid arteries; Psv, peak systolic velocity; Edv, end-diastolic velocity; FGE, fermented garlic extract.

**Table 4:** Comparison of the change in BP and CA velocity before and after FGE intake between the FGE and placebo groups.

	Systolic BP	Diastolic BP	Right				Left			
			CCA		ICA		CCA		ICA	
			Psv	Edv	Psv	Edv	Psv	Edv	Psv	Edv
Z	−2.263	−2.637	−0.874	−2.167	−1.921	−1.595	−0.961	−2.065	−1.267	−0.460
*p*	* 0.024	* 0.008	0.382	* 0.030	0.055	0.111	0.336	* 0.039	0.205	0.645

* *p* < 0.05. BP, blood pressure; CCA, common carotid arteries; ICA, internal carotid arteries; Psv, peak systolic velocity; Edv, end-diastolic velocity.

**Table 5:** Cortex areas showing upregulation of rCBF in the FGE group in comparison to the placebo group after FGE intake.

Coordinates (MNI)			Peak Region	Brodmann Area	*t*	*p _FWE-corr_*
x	Y	z				
+34	−4	+68	Rt. Frontal cortex	6	4.145841	0.00016
+38	+2	+64	Rt. Frontal cortex	6	4.083884	0.000188
+30	−32	+72	Rt. Parietal cortex	2	3.902315	0.000301
+46	+54	+14	Rt. Frontal cortex	10	3.960457	0.000259
+50	+52	+6	Rt. Frontal cortex	10	3.937369	0.000275
+46	+48	+24	Rt. Frontal cortex	9	3.923447	0.000285
−32	+10	+64	Lt. Frontal cortex	6	3.734121	0.000466
−26	+26	+58	Lt. Frontal cortex	6	3.674036	0.000544
−28	+18	+62	Lt. Frontal cortex	6	3.641479	0.000591

MNI, Montreal Neurological Institute; Rt., right; Lt., left; rCBF, regional cerebral blood flow; FGE, fermented garlic extract.

## Data Availability

The data are not publicly available due to privacy of participants.
